# A novel technique for safe intraoperative perilymph sampling in humans

**DOI:** 10.1007/s00405-026-10256-2

**Published:** 2026-06-10

**Authors:** William Shute, Dongcheng Zhang, Amy Hampson, Jean-Marc Gerard, Stephen J. O’Leary

**Affiliations:** Department of Otolaryngology, The University of Melbourne, 32 Gisborne St, East Melbourne, 3002 Australia

**Keywords:** Perilymph sampling, Hearing loss, Human research, Electrocochleography, Cochlear implantation

## Abstract

**Purpose:**

To calibrate nylon epidural catheters against glass capillaries for obtaining reliable volume yields and present the development and implementation of a translatable human perilymph sampling methodology which minimally impacts cochlear function.

**Methods:**

Two gauges of nylon epidural catheters were calibrated against glass capillary tubes to establish the minimum fluid column height to yield a desired sample volume. We also report development of a proposed surgical methodology for in vivo sampling, and implementation in a clinical cohort study of cochlear implant recipients. We assessed the impact of sampling on hearing in a subset of hearing preservation candidates.

**Results:**

Correlation equations for both 18G and 16G catheters yielding a desired sample volume ‘x’ with a fluid column height ‘y’. The equations for 18G and 16G catheters respectively are y = 6.538x + 1.0894 and 3.8008x + 0.6754. A sampling apparatus consisting of an epidural catheter passed through a house suction tip attached to a 1 mL syringe provides optimal ergonomics and manoeuvrability. Extracochlear ECochG amplitudes and thresholds are unaffected by collection of perilymph using this technique, as were 3-month hearing preservation outcomes.

**Conclusion:**

These results describe a safe, accurate and transferrable alternative to using glass capillaries to sample directly from the human inner ear during surgery.

## Introduction

Existing diagnostics in sensorineural hearing loss are deductive and give little characterisation of the underlying nature of the diseased cochlea. As such, available means of hearing rehabilitation must be applied non-discriminately and are limited to medical devices that stimulate the diseased cochlea (i.e. hearing aids and cochlear implants). In order to move towards targeted treatments, novel diagnostics must elicit clinically meaningful information about the deficits in the neuroepithelial sensory apparatus that underlie hearing disability. Sampling of perilymph, the specialised fluid that bathes the membranous labyrinth within the temporal bone, holds promise as such a diagnostic [1–3]. Analysis of perilymph has yielded associations with diagnosis, residual neuroepithelial function and clinical performance with cochlear implantation [4–10]. Otoprotective and regenerative drugs are emerging as treatments for hearing loss. The development and implementation of these therapies relies on a clearer understanding of the biological milieu within the diseased cochlea. Perilymph sampling provides an opportunity to understand the fundamental mechanisms of cochlear dysfunction, confirm drug bioavailability within the cochlea and predict whether adjuvant therapies may be beneficial following cochlear implantation [11, 12].

There is an expanding body of literature involving human perilymph sampling in hearing research; however, the required expertise is limited to just a few institutions. There is a need for a straightforward, reliable and transferrable sampling methodology. Perilymph sampling is an invasive procedure, nonetheless, it has been undertaken without adversely affecting hearing in patients undergoing stapedectomy and cochlear implantation [13]. Published techniques involve either puncture of the round window membrane with a sharpened glass capillary or similar sampling at the oval window via a fenestrated stapes footplate [5, 14, 15]. The use of capillary action is preferable when there is residual hearing function to be preserved. However, the use of glass capillaries raises issues of transferability across jurisdictions. Glass capillaries are not designed for human biospecimen collection nor to withstand the sterilisation procedures that make them appropriate for intraoperative sampling. Glass capillaries may shatter, and their use in the confined bony spaces of the round window niche pose an unnecessary risk to patients. In addition, they are not readily available in clinical settings and are not part of the day-to-day consumables used by ENT surgeons who are required to carry out the sampling procedure.

We set out to validate a safe and effective method of sampling perilymph in humans utilising sterile, single use nylon epidural catheters. Such equipment is readily available in any well stocked operating theatre. However, epidural catheters do not possess appropriate incremental volumetric markings, and their flexibility hampers the surgeon’s ability to manoeuvre the catheter into the round window niche [16, 17]. Our aim is to describe a method that could be easily taken up in any jurisdiction by surgeons previously unfamiliar with perilymph sampling. Herein, we calibrate two sizes of epidural catheter against glass capillaries to determine the minimum draw height to yield a desired volume of perilymph for final biochemical analysis. We present a surgical methodology developed in the pre-clinical and clinical setting to optimise ergonomics and repeatability. Finally, we report the impacts of our sampling method on hearing in hearing preservation cochlear implant candidates in a prospective clinical study. Our method can be used to reliably and repeatedly draw a desired volume of perilymph suitable for analysis, without adversely impacting residual cochlear function.

## Methods

### Epidural catheters

Bench testing was completed using Portex epidural catheters manufactured by Smiths Medical (CE registered number 2797). Two different gauges of catheter were compared to determine which may be more suitable in the surgical setting:

- Epidural catheter 16G clear, closed end 3 eyes, inner Ø 0.55 mm, outer Ø 1.03 (Smiths Medical 100/382/116).
- Epidural catheter 18G clear, closed end 3 eyes, inner Ø 0.45 mm, outer Ø 0.83 (Smiths Medical 100/382/118).

Selection of these gauges was based upon the maximum outer diameter that would be expected to pass through the facial recess and sample fluid from the round window niche in human temporal bones [18]. Nylon catheters were selected over polyurethane catheters as they have a hydrophilic contact angle comparable to that of a glass capillary [19, 20].

### Catheter calibration

Artificial perilymph was used to calibrate the epidural catheters against glass capillary pipettes with 1 µL markers (Blaubrand intraMARK 1–5 µL micropipettes, cat. No. 708707, BRAND, Wertheim, Germany). With the use of a 1 mm incremented ruler capillary volumes as low as 0.07 µL could be quantified. The artificial perilymph solution mimics the ionic composition of human perilymph and exhibits comparable fluid mechanics.

Experiments were devised to determine the fluid column height in mm within an epidural catheter that would yield a desired sample volume with 99.9% confidence accounting for any sample processing wastage that may occur in the laboratory. In our testing, the criteria for evaluating the success of the epidural catheter sampling method was whether the quantified final volume (i.e. the volume ultimately submitted for analysis) was reliably (with 99.9% confidence) greater than the target volume. The individual experiments to determine this, and their results, are described in ***Appendix A***. The correlation equations (Eq. A.3 and 4) in Appendix A Table 3 and Fig. 3 can be used to determine the epidural catheter fluid column height for any desired final volume yield of perilymph.

###  Surgical method development

**Fig. 1:**
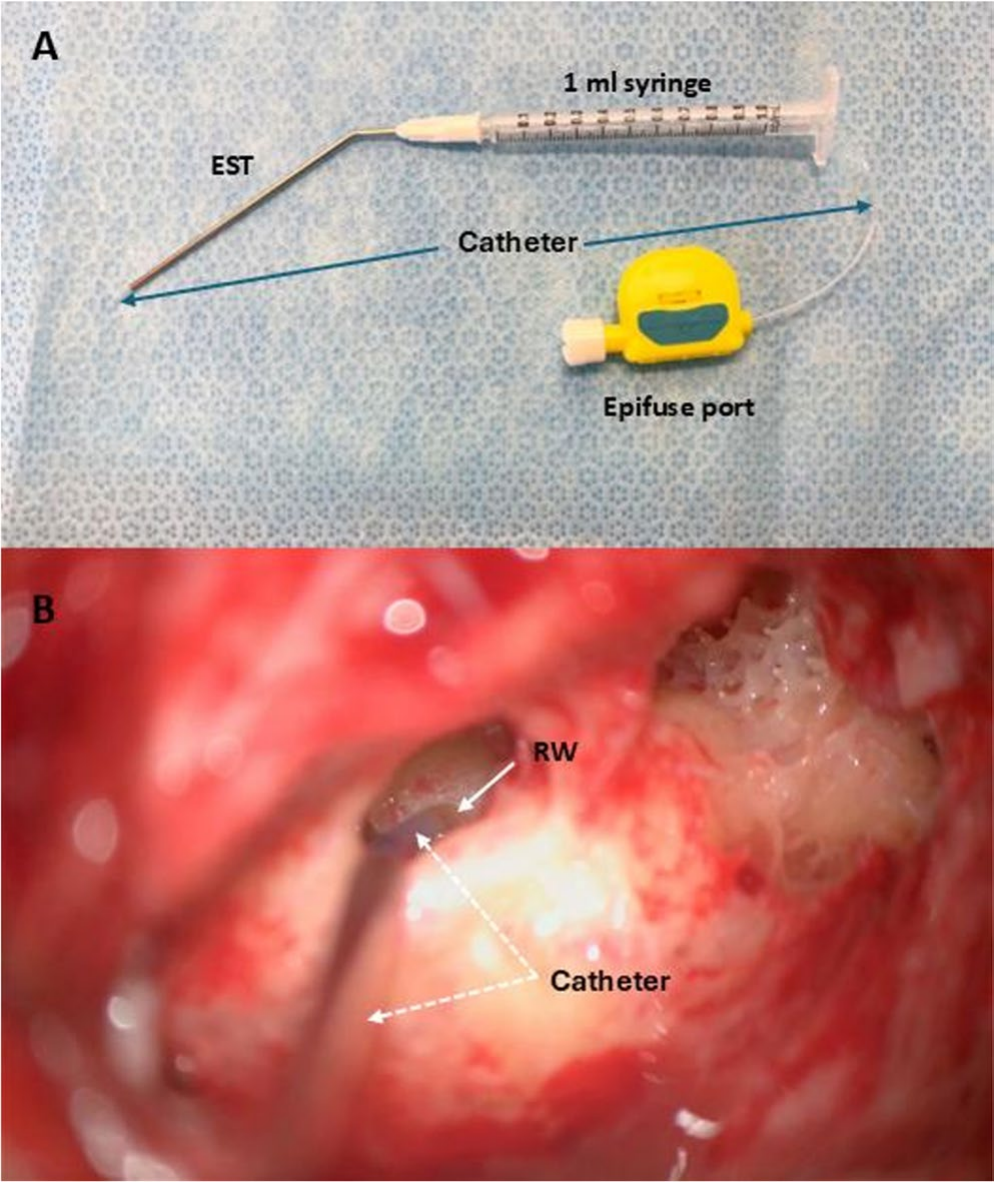
The sampling apparatus and in vivo sampling. **A**). Assembled perilymph sample collection apparatus. This assembly contains catheter, ear suction tip (EST), 1 ml leur lock syringe and ‘Epifuse port’. **B**). In vivo sampling. Epidural sampling catheter in the round window niche (RW)

To devise a surgical methodology for perilymph sampling two cadaveric temporal bones (one right and one left) were drilled by an experienced otologist (JMG). Following removal of soft tissue from the cadaveric bones a standard cortical mastoidectomy and posterior tympanotomy were drilled. The round window overhang was removed with a 1 mm diamond burr to expose the round window membrane which was incised with a 27G hypodermic needle to expose the perilymph of the scala tympani. In order to improve manoeuvrability of the catheter tip the distal (sampling end) of each catheter was passed through the lumen of an appropriately sized Mediplast^®^ “Metallsug” ear suction tip. This in turn was connected to a 1 ml leur lock syringe with the plunger removed to connect the proximal end of the catheter to the ‘Epifuse port’ which allows connection of a 10 ml syringe for an air flush (Fig. 1A).

The sampling apparatus shown in Fig. 1A was assembled with both 16G and 18G catheters. The assembly, as well as the approach to the round window membrane are described in detail in ***Appendix B***. Under microscopic vision both catheters were easily, and with fine manoeuvrability introduced into the round window niche. In both cases the mark indicating desired fluid column heigh was outside of the microscope’s focal width, which needed to be adjusted to confirm the collected sample had reached the required height. While both catheters were able to be used, the 16G catheter was felt to obstruct the operators view of the round window. For this reason, the 18G catheter was thought to be optimal. Uncapping the ‘Epifuse port’ allows sampling via capillary action in situations where hearing preservation is required. Alternatively, in non-hearing preservation cases a 1 ml syringe can be attached to the ‘Epifuse port’ to aspirate a sample. The capillary action draw time reported for the benchtop calibration experiments (Appendix A, Fig. 5) was considerably shorter than in vivo sampling. This relates to surface tension adhesive forces between perilymph and the scala walls, as well as intracochlear pressure. Capillary action sampling took in the order of 2–3 min. This duration emphasises the importance of ensuring rigorous round window niche haemostasis prior to sampling. Additionally, during sampling, a house suction was placed in the mastoid to minimise any accumulation of residual irrigation fluid or blood.

### In-vivo sampling during cochlear implant surgeries

Testing of the methodology in human cochlear implant recipients in the operating theatre (referred to here as ‘in-vivo sampling’) occurred under the auspices of The Royal Children’s Hospital Melbourne HREC, approval number 86,258. Perilymph sampling was undertaken in a population of cochlear implant recipients with a target sample volume of 1 µL. Sampling was preceded by standard exposure of the round window membrane consisting of post-auricular incision, cortical mastoidectomy, posterior tympanotomy and drill-out of the round window overhang. Following exposure of the round window membrane it is essential to attain adequate haemostasis in the soft tissues, mastoid and middle ear such that no blood accumulates in the round window niche when observed for 2 min. This time can be used to assemble the sampling apparatus. A 27G needle on a 1 mL tuberculin syringe was used to incise the round window membrane, this was generally bloodless unless the mucosa overlying the crista finestra was inadvertently incised. Should this occur gentle suction away from the round window alongside a pledget soaked in 1:10000 adrenaline was generally adequate. Once the scala tympani is open the distal end of the sampling catheter is introduced into the round window niche and introduced into the scala through the incision (Fig. 1B). A sample is collected via aspiration or capillary action. The sampling apparatus is then withdrawn, taking care not to contact the bony facial recess which may result in drawing up contaminant, or inadvertent discharge of the specimen. A 10 ml syringe can be connected to the ‘Epifuse port’ to deliver an air flush to discharge the specimen into an Eppendorf tube. The sample immediately undergoes centrifugation in the operating theatre and visually inspected for the presence of a blood pellet at the base of the tube.

### Hearing preservation

Electrocochleography (ECochG) was used to assess the safety of the sampling procedure in a subset of 5 hearing preservation candidates. ECochG traces were acquired using the Cochlear Ltd commercially licenced Apollo program in the Cochlear Research Platform. The cochlear implant’s ball electrode (`Extracochlear Electrode 1`) was conditioned to record and placed on the round window membrane. 500 Hz pure tone pips were presented via an Etymotic Research inc. (Illinois, USA) ER3A silicone sound tube and foam ear tip sited in the subject’s ipsilateral ear canal. The stimulus intensity was set to 100 dB HL and reduced in 5 dB HL increments until no response was obtained. Recordings were made immediately before and after collection of a 1–3 µL perilymph sample.

At our institution, hearing preservation candidates routinely undergo insertion with a slim-straight array (CI622, Cochlear LTD, Sydney, Australia) utilising soft surgical techniques. Additionally, real-time electrode insertion ECochG recording is used, and an ECochG driven feedback intervention protocol as described by Bester et al. is followed [21]. Our extensive experience following up subjects undergoing this ECochG driven protocol allowed us to case-match hearing preservation candidates in this study with those in our database who had not undergone perilymph sampling and compare 3-month hearing preservation results. Case matches were selected on the basis of age at implantation, pre-operative 500 Hz pure tone threshold, and pattern of ECochG response noted during real-time insertion ECochG monitoring (drop in cochlear microphonic amplitude > 30% of previous maximum verses cochlear microphonic amplitude maintained > 30% of previously recorded maximum). 3-month hearing preservation results between cases and controlled were obtained by clinical record audit. Pure tone thresholds at 250 Hz, 500 Hz, 1000 Hz, 2000 Hz and 4000 Hz were compared, along with the low frequency pure tone median (LFPTM) and Skarzynski hearing preservation scores [22]. Additionally, we compared the hearing preservation results with a large unmatched group drawn as a sub-analysis from a recent meta-analysis of the hearing preservation outcomes with the use of intraoperative ECochG testing [23]. This sub-group of 87 patients were selected as they underwent some form of interventional protocol whereby real-time ECochG amplitudes were used to aid minimally traumatic insertion. This reflects the insertion paradigm used at our institution as described above. Statistical analysis was done in Rstudio (Posit, Boston, Massachusetts, USA). Data distribution was assessed using a Shapiro-Wilk normality test. Parametric data was compared using a two-sided t-test while the Wilcoxon rank sum test was used to compare non-parametric data, the Benjamini-Hochberg procedure for false discovery rate correction was used.

## Results

Calibration of both 18G and 16G is described in detail in ***Appendix A***. In brief, for the 18G catheter, deemed most appropriate in wet lab development, the correlation equation describing the fluid column height “x” in mm to yield a desired volume “y” with 99.9% confidence is y = 6.538x + 1.0894 ***(Eq.(A.4))***. For example, if 1 µL of perilymph is required for a desired analysis, 7.6 mm of fluid should be drawn. This accounts for sample processing wastage in the transfer of sample from the catheter to the receptacle in the operating theatre, and from the receptacle to a micropipette in the laboratory. As such, the actual volume yield is approximately 1.38 µL.

**Fig. 2:**
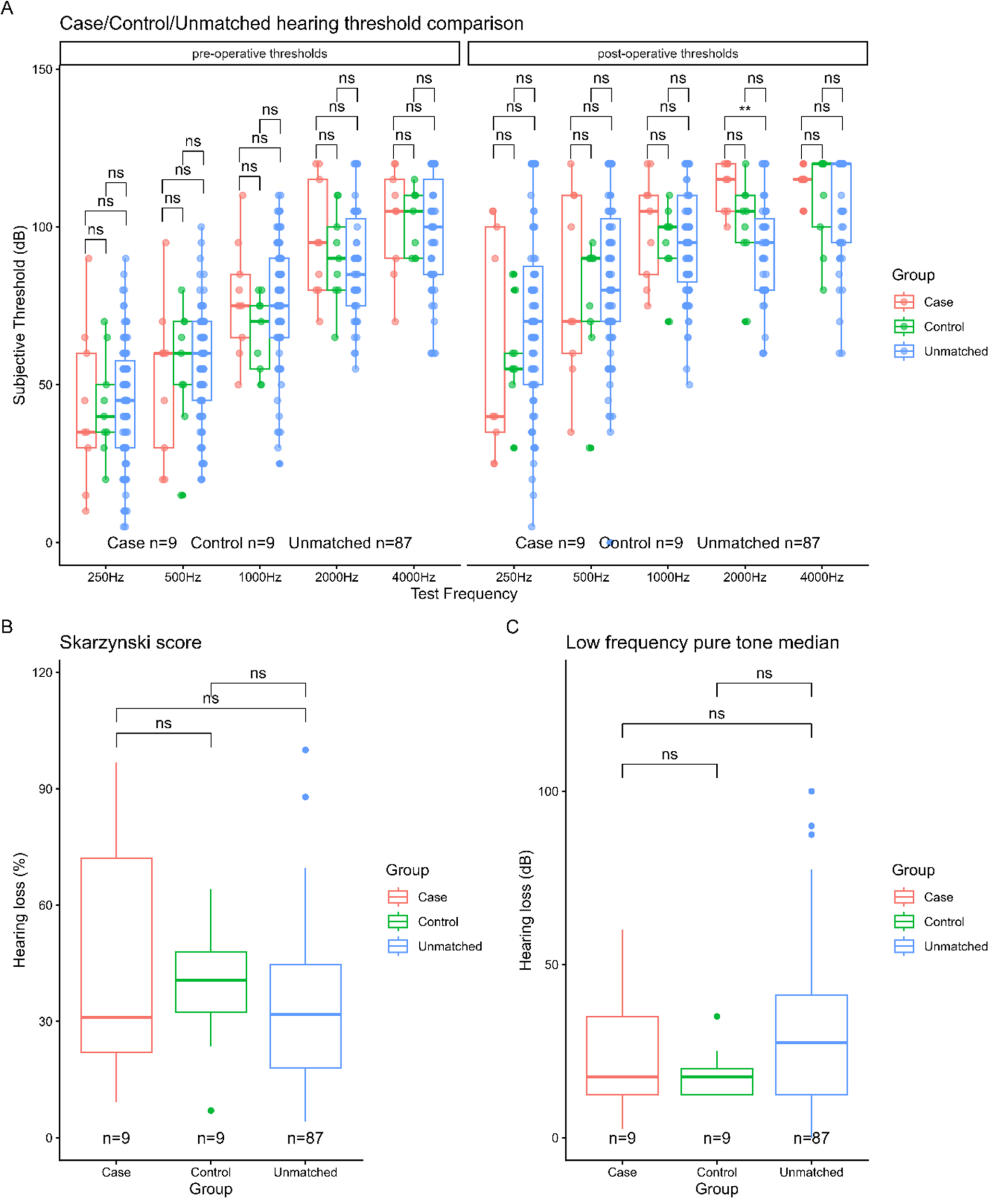
Comparison of 3-month audiometric outcomes in hearing preservation candidates undergoing perilymph sampling with age, audiogram and real-time ECochG matched controls, and a cohort of unmatched CI recipients undergoing electrode insertion under a real-time ECochG drive feedback protocol. **A**). Case/Control hearing threshold comparison; **B**). Skarzynski score. **C**). Low frequency pure tone median

**Fig. 3:**
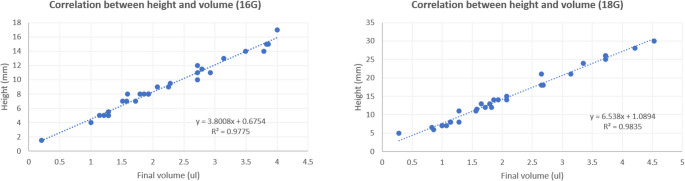
Correlations between fluid heights collected in the catheters and final volumes quantified in the glass capillary pipettes

**Fig. 5:**
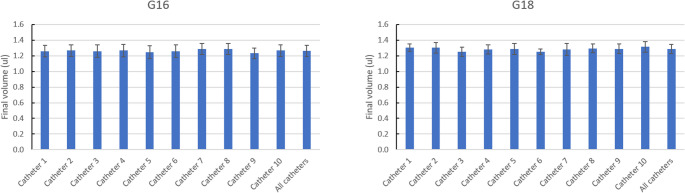
The final volumes gained using the minimum height of fluid in the catheter defined in Experiment 3 (i.e. 5 mm for 16G and 9 mm for 18G catheters). Error bars represent the standard deviation across 10 tests per catheter

In-vivo sampling in 24 subjects was completed with no intraoperative complications or technical failures. 2/24 subjects experienced vertigo in the first post operative week which was treated with a course of oral corticosteroid and resolved. Extracochlear ECochG before and after sampling was carried out in 5 subjects (86258/09, 86258/18, 86258/21, 86258/23 and 86258/24). There was no significant change in cochlear microphonic amplitude in response to a 100 dB HL stimulus (0.67 µV vs. 0.87 µV *p* = 0.2220) and no significant change in cochlear microphonic threshold (78 dB vs. 78 dB, *p* = 0.9990) before and after perilymph sampling. There was a trend for larger cochlear microphonic amplitudes at threshold stimulus intensity, (*p* = 0.0952) which may have related to a widening of the round window membrane incision to facilitate sampling in 2 subjects. Case matches for the 9 hearing preservation candidates are shown in Table 1. Subjects were adequately matched for age (mean age 53.1 vs. 56.4, *p* = 0.8030) pre-operative 500 Hz pure tone threshold (mean threshold 51.1 dB vs. 55.6 dB, *p* = 0.6790) and pre-operative pure tone average (mean PTA = 73.8 dB vs. 71.4 dB, *p* = 0.6240). Demographics of the unmatched cohort are not presented here. Pre- and post-operative hearing thresholds by group are shown in Fig. 2A. The unmatched cohort has a post operative 2000 Hz pure tone threshold of 92.8 dB, which was significantly lower than the case group (113.3 dB, *p* = 0.001). There were no other differences in pre- and post-operative thresholds. The 3-month hearing preservation results are also shown in Fig. 2B and 2C. There was no significant difference in Skarzynski hearing preservation score between groups. The case group had a mean relative loss of 49.6%, which was not different to both the matched control group (mean relative loss 38.8%, *p* = 0.860) and the unmatched controls (relative loss 36.6%, *p* = 0.345). (Fig. 2B). Figure 2C compares the mean LFPTM loss across groups. The LFPTM loss in the case group was 25.0 dB, this was not significantly different to either the matched controls (mean loss 18.6 dB, *p* = 0.857) of the unmatched controls (mean loss 30.1 dB, *p* = 0.596).

**Table 1:** Case-control matching of hearing preservation candidates undergoing perilymph sampling. Cases are matched on age, pre-operative 500 Hz threshold, and whether or not there was a drop in cochlear microphonic amplitude by greater than 30% on real time, electrode insertion ECochG. Subjects who underwent pre- and post- sampling extracochlear ECochG are noted with an ‘*’, note that subject 86,258/23 underwent pre- and post- sampling ECochG, but did not attend their 3-month audiometric evaluation

Identifier	Group	Age	Insertion CM amplitude pattern	PRE-SURGERY Audiometry	
				500hz threshold (dB)	pure tone average (DB)
86,258/03	Case	71	no drop	60	68
Match03(060_SB)	Control	76	no drop	65	76
86,258/04	Case	33	no drop	20	76
Match04(005_JS)	Control	33	no drop	50	79
86,258/05	Case	84	no drop	95	91
Match05(076_RC)	Control	92	no drop	60	68
86,258/06	Case	5	no drop	30	62
Match06(053_TG)	Control	6	no drop	50	65
86,258/09*	Case	35	drop	60	89
Match09(106_MC)	Control	42	drop	70	72
86,258/17	Case	68	no drop	60	76
Match17(064_PC)	Control	67	no drop	70	70
86,258/18*	Case	77	drop	70	68
Match18(129_JC)	Control	78	drop	80	88
86,258/21*	Case	77	drop	20	58
Match21(128_MC)	Control	75	drop	40	63
86,258/24*	Case	28	no drop	45	76
Match24(107_AG)	Control	39	no drop	15	62

## Discussion

The primary aim of this study was to validate the use of single-use, sterile epidural catheters for perilymph sampling in humans, to develop a reliable surgical methodology for this procedure and to present our initial observations on the safety of the procedure in a prospective clinical cohort study. Our results demonstrate that both 18G and 16G nylon epidural catheters can be effectively calibrated against glass capillary tubes to yield precise sample volumes, with the 18G catheter emerging as the optimal choice due to its superior ergonomics and minimal obstruction of the surgical field. The correlation equations derived for these catheters provide a robust framework for ensuring accurate sample collection. The correlation equation for a 16G catheter is y = 3.8008x + 0.6754 ***(Appendix A. Eq.(A.3))***, and the correlation equation for an 18G is y = 6.538x + 1.0894 ***(Appendix A. Eq.(A.4))***. The surgical sampling technique, which is outlined in detail in ***Appendix B***, required minimal training and was easily applied by the experienced otologists working at our institution. We have successfully implemented this method in two separate clinical studies requiring the collection of perilymph prior to insertion of a cochlear implant electrode array. One of these studies allowed us to assess the effect of perilymph sampling on the residual hearing of hearing preservation cochlear implant candidates. Herein, we utilised intraoperative extracochlear ECochG to examine the effect of perilymph sampling on the amplitude and threshold of the cochlear microphonic. Intraoperative extracochlear ECochG has been used to monitor the cochlear microphonic in observational studies examining hearing preservation in cochlear implant surgery [24]. The cochlear microphonic is visualised as an alternating current response phase locked with the stimulus frequency. While it has multiple contributions it is predominated by the outer hair cell response and is a validated surrogate of auditory function [25, 26]. Our results show no reduction in cochlear microphonic amplitude or increase in stimulus threshold after sampling which would suggest a deleterious effect on hearing. These results are expected as small amounts of perilymph are often observed to be lost during stapes surgery for otosclerosis with no deleterious effect on bone conduction thresholds. In the existing literature where glass capillaries are used to sample perilymph no concerns have been raised over poorer hearing preservation outcomes [2, 7–10, 15]. 9/24 subjects in our prospective clinical cohort study were candidates for hearing preservation, providing us with an opportunity to assess the effect of perilymph sampling on their audiological outcomes. We demonstrated that this cohort’s hearing preservation outcomes fell within the expected range based on existing literature [27]. Furthermore, taking advantage of our extensive experience with hearing preservation research, we were able to retrospectively case match these 9 subjects with a cohort with similar, operative and pre-operative demographics. Furthermore, we compared the outcomes with a larger, unmatched cohort drawn from a recent meta-analysis on the relationship between hearing preservation and the use of intraoperative intracochlear ECochG [23]. The comparison of cases with matched and unmatched controls revealed no significant differences in 3-month hearing preservation results. We acknowledge that these represent preliminary results only, and limited by the wide distribution of pre-operative pure tone data presented in Fig. 2A, and that large numbers of subjects would be required to detect small differences in hearing preservation results. However, such small differences would be unlikely to translate into reduced utility of electroacoustic stimulation, poorer speech reception or poorer quality of life outcomes.

Compared with existing studies which utilise modified glass capillaries, our method utilises equipment available in any operating theatre and familiar to all clinical staff [2, 28]. In developing this technique, we aim to make human perilymph sampling research more accessible to academic otology units, and more palatable to Human Research and Ethics Committees. The key pitfalls and learnings we have undertaken through multiple clinical trials applying this technique include: Meticulous exposure of the round window niche including removal of residual irrigation fluid and blood in the mastoid, round window membrane incision spanning the full height of the membrane, a single pass of the catheter into the round window niche (as a pose to multiple withdrawals to check the height of the fluid meniscus) and undertaking the initial sample processing steps of centrifugation and snap freezing immediately in the operating theatre. Following these steps this technique is a user friendly, safe, reliable and transferrable methodology for sampling perilymph in humans.

## Conclusion

Human perilymph sampling research is expanding understanding of which biological processes in the inner ear are implicated in hearing loss and contribute to hearing rehabilitation outcomes. With the advent of inner ear therapeutics, the potential to better understand the mechanisms of hearing loss through human perilymph analysis is of great interest. Currently, expertise in human perilymph sampling is not widespread, limiting the uptake of this research. Our method represents a transferrable, reliable and safe means of sampling human perilymph, achievable by any experienced otologist.

## Figures and Tables

**Fig. 4:**
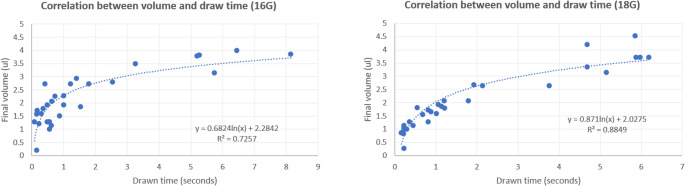
The relationship between the final volumes quantified in the glass capillary pipettes and the time taken to draw up fluid in the catheters

## Appendix (supplementary) A – benchtop calibration of epidural catheters against glass capillaries

### Experiment 1 – Estimate the catheter internal diameter and evaluate its variation

Experiment 1 aimed to estimate the variation between catheter internal diameter. For each catheter gauge 3 µL of artificial perilymph was aliquoted into 110 Eppendorf tubes using adjustable micro-pipettes. The fluid was then drawn from these tubes via capillary action using either a glass capillary pipette (10 tubes; 10 glass capillary pipettes) or the catheters (100 tubes; 10 different catheters 10 times each). The fluid height in the catheters and the volume collected in the glass pipettes was recorded for each sample drawn up. The internal diameter for each catheter was then estimated using the below formula, derived from the formula for the volume of a cylinder:

A.1 $$\begin{array}{c}Internal\;diameter=\\2\surd\frac{mean\;volume\;\in\;glass\;pipette}{\mathrm\pi\;\left(\mathrm{mean}\;\mathrm{height}\;\in\;\mathrm{catheter}\right)}\end{array}$$

## Results

When drawing up an equal volume of fluid, there was very little variation in fluid height between catheters. For 16G catheters the mean height was 10.76 mm (SD= 0.37) and for 18G catheters the mean height was 19.75 mm (SD = 0.34), with all measurements being within a 1 mm range for both gauges. The calculated internal diameters for each catheter are presented in Table 2.

**Table 2:** Mean volumes (µL) in glass pipettes and mean height (mm) of fluid in the catheters were used to calculate the estimated internal diameter of the catheters

Catheter type	Volume in glass pipette $\left(\mu L\right)$		Height in catheters (mm)		Estimated internal diameters (mm)
	Mean	SD	Mean	SD	
16G	2.92	0.06	10.76	0.37	0.59
18G	3.02	0.03	19.75	0.34	0.44

### Experiment 2 – establish correlations between the final volume collected and the fluid height in the catheter

Experiment 2 aimed to determine correlations between the *final volume* collected and a) the fluid height in the catheter and b) the time required to draw the fluid into the catheter. A random volume of fluid was drawn into each catheter, and the fluid height and draw time were recorded. The fluid was then discharged into an empty 0.5 mL Eppendorf tube and redrawn with a glass pipette to quantify the final volume collected. This was repeated three times for each catheter (i.e. 30 times in total for each gauge). The results were then graphed to establish correlations. These correlations were used to determine an *estimated height* of fluid drawn into the catheters used in Experiment 3.

## Results

The fluid height in the catheters had a strong linear correlation with the final volume quantified in the glass capillary pipettes (R^2^= 0.9775 and 0.9835 for the 16G and 18G catheters respectively; Fig. 3). The correlation equation for the 16G catheters was determined to be y= 3.8008x + 0.6754, giving an estimated height of 4.48 mm to collect a final volume of 1 µL. The correlation equation for the 18G catheters was determined to be y= 6.538x + 1.0894, giving an estimated height of 7.63 mm to collect a final volume of 1 µL. Therefore, the heights of 4 mm for 16G and 8 mm for 18G were used as the estimated heights in Experiment 3 to establish the 99.9% confidence interval.

Whilst the relationship between the final volume quantified in the glass capillary pipettes and the time taken to draw fluid up was not linear, it fit a logarithmic curve well (Fig. 4). Fluid was able to be drawn up quickly at first, but over time the rate of capillary action decreased, and the volume increased slowly.

### Experiment 3 – determine the minimum height of fluid drawn into the catheter to have ≥1 µL perilymph available for analysis

Experiment 3 aimed to establish the *minimum height* (in mm) of fluid drawn into the catheter to have ≥1 µL perilymph available for analysis, with a confidence interval of 99.9%. To achieve this, fluid was drawn into the catheters to the *estimated height* established in Experiment 2 using a desired final volume of ≥1 µL. The fluid was then discharged into a glass capillary pipette and the final volume in the pipette was recorded. This process was repeated with each individual catheter (i.e. 10 of each gauge).

The mean final volume and the standard deviation (SD) was used to determine the minimum mean amount of fluid required to meet the 99.9% confidence interval (i.e. mean +3SD). This minimum mean served as the required final volume, and it was used with the correlation equations established in Experiment 2 to calculate the minimum height of fluid drawn into the catheter to have ≥1 µL perilymph available with a confidence interval of 99.9%. As noted previously, the final volume redrawn from the Eppendorf tubes (step 4) was less than the collected volume in the catheters (step 2) due to processing loss. Once the minimum height of fluid drawn to obtain the required final volume was determined, we also estimated the original collected volume using the catheters inner diameter (obtained in Experiment 1) and the below equation, derived from the formula for the volume of a cylinder:

A.2 $$\begin{array}{c}\mathrm{Estimated}\;\mathrm{collected}\;\mathrm{volume}=\\\mathrm\pi\left(\mathrm{minimum}\;\mathrm{height}\;\in\;\mathrm{catheter}\right)\left(\frac{\mathrm{inner}\;\mathrm{diameter}\;\mathrm{of}\;\mathrm{catheter}}2\right)^2\end{array}$$

## Results

Drawing fluid up to the estimated height of 4 mm for the 16G catheters gave a mean final volume of 1.06 µL with a SD of 0.05. For the 18G catheters, drawing fluid up to the estimated height of 8 mm gave a mean final volume of 1.15 µL with a SD of 0.06. These standard deviations were used to calculate the minimum mean required to obtain a desired final volume. These results are summarised in Table 2 using the indicative volume of 1 µL.

### Experiment 4 – evaluate the reliability of the defined minimum height

Experiment 4 aimed to evaluate the reliability of the defined minimum height. To achieve this fluid was drawn into the catheters to the minimum height defined in Experiment 3, and the time taken to reach this height was recorded. The fluid was then discharged into an 0.5 mL Eppendorf tube and redrawn into a glass capillary pipette. The volume of fluid in the glass capillary pipette was then recorded. This process was repeated 10 times for each individual catheter.

## Results

Here the minimum heights defined in Experiment 3 (i.e. 5 mm for 16G and 9 mm for 18G catheters) were evaluated for reliability. Under the defined minimum heights, there was no difference in the final volumes gained among the 16G or 18G catheters, respectively (Fig. 5). Tables 3 and 4 summarises the mean final volumes, SD, 99.9% CI, and minimum and maximum final volumes for each tested catheter. Under the defined minimum height, the 99.9% CI is greater than 1 µL for all tested catheters and there was no single test where the final volume was below 1 µL (Table 5).

**Table 3:** Summary of experiment 3 results

Catheter type	16G	18G
Correlation equation	Y = 3.8008x + 0.6754 Eq.(A.3)	Y = 6.538x + 1.0894 Eq.(A.4)
Estimated height (mm) in catheter for 1 µL final volume	4	8
Mean final volume (µL) using estimated height	1.06	1.15
SD of mean final volume	0.05	0.06
99.9% CI (µL)	0.91	0.96
Minimum mean final volume required to fulfil the 99.9% CI (µL)	1.15	1.18
Minimum height in catheter to fulfil the 99.9%CI (mm)	**5**	**9**
Estimated internal diameter (mm, from Experiment 1)	0.59	0.44
Estimated volume that will be drawn from the cochlea to obtain a final volume of ≥ 1 µL with 99.9%CI (µL)	**1.36**	**1.38**

**Table 4:** Summary of mean final volumes, SD, 99.9% CI and minimum and maximum final volumes across 10 tests for each 16G catheter when drawing fluid up to the defined minimum height of 5 mm. All volumes are given in µL

16G	Catheter 1	Catheter 2	Catheter 3	Catheter 4	Catheter 5	Catheter 6	Catheter 7	Catheter 8	Catheter 9	Catheter 10	All Catheters
Mean	1.26	1.27	1.26	1.27	1.25	1.26	1.29	1.29	1.23	1.27	1.26
SD	0.07	0.07	0.08	0.08	0.08	0.08	0.07	0.07	0.07	0.07	0.07
99.9% CI	1.04	1.05	1.02	1.03	1.00	1.02	1.08	1.08	1.03	1.05	1.04
Min	1.21	1.21	1.14	1.21	1.14	1.21	1.14	1.21	1.21	1.14	1.14
Max	1.35	1.35	1.35	1.35	1.35	1.28	1.35	1.35	1.35	1.35	1.35

**Table 5:** Summary of mean final volumes, SD, 99.9% CI and minimum and maximum final volumes across 10 tests for each 18G catheter when drawing fluid up to the defined minimum height of 5 mm. All volumes are given in µL

18G	Catheter 1	Catheter 2	Catheter 3	Catheter 4	Catheter 5	Catheter 6	Catheter 7	Catheter 8	Catheter 9	Catheter 10	All Catheters
Mean	1.30	1.30	1.25	1.28	1.29	1.25	1.28	1.29	1.29	1.32	1.28
SD	0.05	0.07	0.06	0.06	0.07	0.04	0.07	0.06	0.06	0.07	0.06
99.9% CI	1.16	1.10	1.07	1.11	1.08	1.14	1.06	1.13	1.10	1.11	1.10
Min	1.21	1.21	1.14	1.21	1.14	1.21	1.14	1.21	1.21	1.14	1.14
Max	1.35	1.35	1.35	1.35	1.35	1.28	1.35	1.35	1.35	1.35	1.35

## Appendix (supplementary) B – perilymph sampling surgical manual

### Required equipment

1. An epidural catheter kit (Portex 100/382/118) Gauge #18 including an epidural catheter, Epifuse® port and a luer lock syringe.
2. A 0.5 mL Eppendorf tube.
3. A#15 scalpel blade.
4. A fresh dissection block.
5. A sterile surgical ruler with 1 mm graduations.
6. A fine forceps such as a fine jeweller’s forceps or an electrode insertion forceps.
7. A long 25G or 27G disposable hypodermic needle for incising the round window membrane, as per the surgeon’s preference.
8. A 17G disposable ENT metal suction tube (e.g Mediplast® Metalsug suction tube).
9. Two 1 ml tuberculin syringes, one 10 ml luer slip syringe.

### Assembly of sampling apparatus

1. The distal end (sampling end) of the catheter is trimmed using the blade on the cutting block to remove the three lateral eye channels.
2. A surgical marker is used to mark the distal end of the catheter at the height which corresponds to the desired sample yield.
3. The distal end of the catheter is passed through the lumen of a 17G Mediplast® “Metallsug” ear suction tip. This in turn was connected to a 1 ml leur lock syringe with the plunger removed.
4. The proximal end of the catheter is inserted into the ‘Epifuse port’ (Fig. 1).

### Sampling procedure

1. The round window is exposed in the routine surgical fashion.
2. The middle ear and mastoid are cleared of blood and irrigant to minimise the risk of sample contamination.
3. An incision the full length of the round window membrane is made with a 25G or 27G needle.
4. The distal tip of the catheter is advanced through the facial recess to the round window niche, such that the mark denoting the desired collection height is visible.
5. The distal tip of the sampling catheter is introduced into the scala tympani and the fluid meniscus is observed until it reaches the mark.
6. Withdraw the sampling apparatus, taking care not to contaminate the sample with blood or irrigant from the mastoid.
7. Place the distal tip of the sampling catheter into an appropriately sized Eppendorf tube.
8. Attach a 10 mL syringe to the Epifuse port and deliver a 10 ml air flush to discharge the sample into the Eppendorf tube.

## References

[1] Alawieh A et al (2015) Proteomics studies in inner ear disorders: pathophysiology and biomarkers. Expert Rev Proteomics 12(2):185–196 25795149 10.1586/14789450.2015.1024228

[2] Peter MS, Warnecke A, Staecker H (2022) A window of opportunity: perilymph sampling from the round window membrane can advance inner ear diagnostics and therapeutics. J Clin Med. 10.3390/jcm11020316 35054010 10.3390/jcm11020316 PMC8781055

[3] Salt AN, Hale SA, Plonkte SK (2006) Perilymph sampling from the cochlear apex: a reliable method to obtain higher purity perilymph samples from scala tympani. J Neurosci Methods 153(1):121–129 16310856 10.1016/j.jneumeth.2005.10.008 PMC1769328

[4] Chen HHR, Wijesinghe P, Nunez DA (2019) MicroRNAs in acquired sensorineural hearing loss. J Laryngol Otol 133(8):650–657 31358070 10.1017/S0022215119001439

[5] de Vries I et al (2019) Detection of BDNF-related proteins in human perilymph in patients with hearing loss. Front Neurosci 13:214 30971872 10.3389/fnins.2019.00214 PMC6445295

[6] Wichova H, Shew M, Staecker H (2019) Utility of perilymph microRNA sampling for identification of active gene expression pathways in otosclerosis. Otol Neurotol 40(6):710–719 31192899 10.1097/MAO.0000000000002243

[7] Shew M (2021) MicroRNA profiling as a methodology to diagnose Meniere’s Disease: potential application of machine learning. Otolaryngol Head Neck Surg 164(2):399–406 32663060 10.1177/0194599820940649 PMC12375223

[8] Shew M (2021) Distinct microRNA profiles in the perilymph and serum of patients with Meniere’s Disease. Front Neurol 12:646928 34220670 10.3389/fneur.2021.646928 PMC8242941

[9] Shew M et al (2021) Evaluating Neurotrophin Signaling Using MicroRNA Perilymph Profiling in Cochlear Implant Patients With and Without Residual Hearing. Otol Neurotol 42(8):e1125–e1133 33973949 10.1097/MAO.0000000000003182

[10] Durisin M et al (2022) Proteome profile of patients with excellent and poor speech intelligibility after cochlear implantation: can perilymph proteins predict performance? PLoS One 17(3):e0263765 35239655 10.1371/journal.pone.0263765 PMC8893673

[11] Patel M, Hu BH (2012) MicroRNAs in inner ear biology and pathogenesis. Hear Res 287(1–2):6–14 22484222 10.1016/j.heares.2012.03.008 PMC3358572

[12] Rudnicki A, Avraham KB (2012) microRNAs: the art of silencing in the ear. EMBO Mol Med 4(9):849–859 22745034 10.1002/emmm.201100922 PMC3491818

[13] Schmitt HA et al (2017) Proteome analysis of human perilymph using an intraoperative sampling method. J Proteome Res 16(5):1911–1923 28282143 10.1021/acs.jproteome.6b00986

[14] Edvardsson Rasmussen J et al (2018) The proteome of perilymph in patients with vestibular schwannoma. A possibility to identify biomarkers for tumor associated hearing loss? PLoS One 13(6):e0198442 29856847 10.1371/journal.pone.0198442 PMC5983529

[15] Shew M et al (2018) Feasibility of microRNA profiling in human inner ear perilymph. Neuroreport 29(11):894–901 29781875 10.1097/WNR.0000000000001049

[16] Mehanna AM, Abdelnaby MM, Eid M (2020) The anatomy and anatomical variations of the round window prechamber and their implications on cochlear implantation: an anatomical, imaging, and surgical study. Int Arch Otorhinolaryngol 24(03):e288–e298 32754239 10.1055/s-0039-1698783 PMC7394623

[17] Mehanna AM, Abdelnaby MM, Eid M (2020) The anatomy and anatomical variations of the round window prechamber and their implications on cochlear implantation: an anatomical, imaging, and surgical study. Int Arch Otorhinolaryngol 24:288–298 10.1055/s-0039-1698783 PMC7394623 32754239

[18] Jain S et al (2019) Anatomical Study of the Facial Recess with Implications in Round Window Visibility for Cochlear Implantation: Personal Observations and Review of the Literature. Int Arch Otorhinolaryngol 23(3):e281–e291 31360247 10.1055/s-0038-1676100 PMC6660289

[19] Al-Zaidi E, Fan X (2018) Effect of aqueous electrolyte concentration and valency on contact angle on flat glass surfaces and inside capillary glass tubes. Colloids Surf A Physicochem Eng Asp 543:1–8

[20] Tokoro T, Hackam R (1995) Effects of water salinity, electric stress and temperature on the hydrophobicity of nylon. in Proceedings of 1995 Conference on Electrical Insulation and Dielectric Phenomena. IEEE

[21] Bester C et al (2021) Electrocochleography triggered intervention successfully preserves residual hearing during cochlear implantation: Results of a randomised clinical trial. Hear Res 108353 10.1016/j.heares.2021.108353 34600798

[22] Skarzynski H et al (2013) Towards a consensus on a hearing preservation classification system. Acta Otolaryngol Suppl (564):3–13 10.3109/00016489.2013.869059 24328756

[23] Samuel O et al (2025) Intraoperative Intracochlear Electrocochleography and Hearing Preservation Early after Cochlear Implantation: A Meta-Analysis. Audiology and Neurotology 10.1159/000549281 41317334

[24] Dalbert A et al (2015) Extra- and intracochlear electrocochleography in cochlear implant recipients. Audiol Neurootol 20(5):339–348 26340649 10.1159/000438742

[25] Ferraro JA, City K (2000) Clinical electrocochleography: overview of theories, techniques and applications. Audiology Online

[26] Forgues M et al (2014) Distinguishing hair cell from neural potentials recorded at the round window. J Neurophysiol 111(3):580–593 24133227 10.1152/jn.00446.2013 PMC3921406

[27] Santa Maria PL et al (2014) Hearing preservation surgery for cochlear implantation: a meta-analysis. Otology Neurotology 35(10):e256–e269 25233333 10.1097/MAO.0000000000000561

[28] Warnecke A et al (2019) Defining the inflammatory microenvironment in the human cochlea by perilymph analysis: Toward liquid biopsy of the cochlea. Front Neurol 10:665 31293504 10.3389/fneur.2019.00665 PMC6603180

